# Global Marine Cold Seep Metagenomes Reveal Diversity of Taxonomy, Metabolic Function, and Natural Products

**DOI:** 10.1093/gpbjnl/qzad006

**Published:** 2023-12-13

**Authors:** Tao Yu, Yingfeng Luo, Xinyu Tan, Dahe Zhao, Xiaochun Bi, Chenji Li, Yanning Zheng, Hua Xiang, Songnian Hu

**Affiliations:** State Key Laboratory of Microbial Resources, Institute of Microbiology, Chinese Academy of Sciences, Beijing 100101, China; University of Chinese Academy of Sciences, Beijing 100049, China; State Key Laboratory of Microbial Resources, Institute of Microbiology, Chinese Academy of Sciences, Beijing 100101, China; University of Chinese Academy of Sciences, Beijing 100049, China; State Key Laboratory of Microbial Resources, Institute of Microbiology, Chinese Academy of Sciences, Beijing 100101, China; University of Chinese Academy of Sciences, Beijing 100049, China; State Key Laboratory of Microbial Resources, Institute of Microbiology, Chinese Academy of Sciences, Beijing 100101, China; University of Chinese Academy of Sciences, Beijing 100049, China; State Key Laboratory of Microbial Resources, Institute of Microbiology, Chinese Academy of Sciences, Beijing 100101, China; University of Chinese Academy of Sciences, Beijing 100049, China; State Key Laboratory of Microbial Resources, Institute of Microbiology, Chinese Academy of Sciences, Beijing 100101, China; University of Chinese Academy of Sciences, Beijing 100049, China; State Key Laboratory of Microbial Resources, Institute of Microbiology, Chinese Academy of Sciences, Beijing 100101, China; University of Chinese Academy of Sciences, Beijing 100049, China; State Key Laboratory of Microbial Resources, Institute of Microbiology, Chinese Academy of Sciences, Beijing 100101, China; University of Chinese Academy of Sciences, Beijing 100049, China; State Key Laboratory of Microbial Resources, Institute of Microbiology, Chinese Academy of Sciences, Beijing 100101, China; University of Chinese Academy of Sciences, Beijing 100049, China

**Keywords:** Global marine cold seep, Metagenome, Prokaryotic microbiome, Metabolic function, Natural product

## Abstract

Cold seeps in the deep sea are closely linked to energy exploration as well as global climate change. The alkane-dominated chemical energy-driven model makes cold seeps an oasis of deep-sea life, showcasing an unparalleled reservoir of microbial genetic diversity. Here, by analyzing 113 metagenomes collected from 14 global sites across 5 cold seep types, we present a comprehensive Cold Seep Microbiomic Database (CSMD) to archive the genomic and functional diversity of cold seep microbiomes. The CSMD includes over 49 million non-redundant genes and 3175 metagenome-assembled genomes, which represent 1895 species spanning 105 phyla. In addition, beta diversity analysis indicates that both the sampling site and cold seep type have a substantial impact on the prokaryotic microbiome community composition. Heterotrophic and anaerobic metabolisms are prevalent in microbial communities, accompanied by considerable mixotrophs and facultative anaerobes, highlighting the versatile metabolic potential in cold seeps. Furthermore, secondary metabolic gene cluster analysis indicates that at least 98.81% of the sequences potentially encode novel natural products, with ribosomally synthesized and post-translationally modified peptides being the predominant type widely distributed in archaea and bacteria. Overall, the CSMD represents a valuable resource that would enhance the understanding and utilization of global cold seep microbiomes.

## Introduction

Marine cold seeps are special chemoenergetic trophic ecosystems driven by gaseous and liquid hydrocarbons from deep geologic sources [1,2]. Despite such extreme environmental conditions of low oxygen and temperature, high pressure, and absence of light [3], the anaerobic methanotrophic archaea (ANME) and sulfate-reducing bacteria (SRB) are dominant with the utilization of methane and other alkanes [4–7]. Methane-dominated short-chain alkanes released from cold seeps may enter the atmosphere and thus affect the global climate, accompanied by natural leakage processes and human mining activities [8]. In addition, mining activities may negatively affect biodiversity at regional and global scales by disrupting the original microbial communities of cold seeps [9]. Therefore, understanding the microbiome composition associated with cold seeps is critical for addressing the global energy crisis and climate change, as well as for utilizing the microbial resources of cold seeps.

In recent years, with advances in high-throughput sequencing technologies and computational methods, several comprehensive metagenomic databases have been constructed, including glacier [10], marine [11], human [12], and Earth [13] microbiomes. These studies have contributed to a substantial understanding of microbial community composition and metabolic properties of microbiomes in specific habitats. Although there are studies related to the microbial community composition [2,14,15], carbon cycling [5,7,16–18], and nitrogen cycling [19,20] in cold seeps, a comprehensive and complete database integrating all known global cold seep samples remains unavailable. This inevitably limits the systematic understanding of cold seep microbiomes.

Furthermore, because cold seeps possess a rich species diversity and the vast majority are uncultured, they may harbor tremendous phylogenetic, metabolic, and functional diversity. Natural products produced by diverse secondary metabolite biosynthetic gene clusters (BGCs) mainly include the non-ribosomal peptide synthetases (NRPSs), polyketide synthases and their derivatives (PKSI, PKSII, and PKS other), PKS–NRPS hybrids, ribosomally synthesized and post-translationally modified peptides (RiPPs), and terpenes [21]. It has been widely demonstrated to have substantial value in medicine, agriculture, and biotechnology [22,23]. For example, from 1981 to 2019, 36.3% of new drugs approved by the US Food and Drug Administration are natural products or their derivatives [22]. Numerous studies have shown that a large number of uncultured microbiomes encoding BGCs exist in land [13], marine [11,13], and glacier [10] environments. However, the biosynthetic potential of cold seep microbiomes remains largely unexplored.

Currently, scattered and non-uniform metagenomic studies limit the understanding of microbial diversity in the global cold seep ecosystem. Accordingly, we performed an integrative analysis of 113 metagenomes from 14 global sites covering 5 cold seep types. Here, we present the prokaryote-focused Cold Seep Microbiomic Database (CSMD). The catalog includes 1895 potential species-level prokaryotic genomes derived from 3175 non-redundant metagenome-assembled genomes (MAGs), 27 million contigs, and over 49 million non-redundant genes, thus facilitating the exploration of global cold seep microbial composition and metabolic diversity, as well as the assessment of natural product synthetic potential in particular.

## Results

### Construction of CSMD

**Figure 1:**
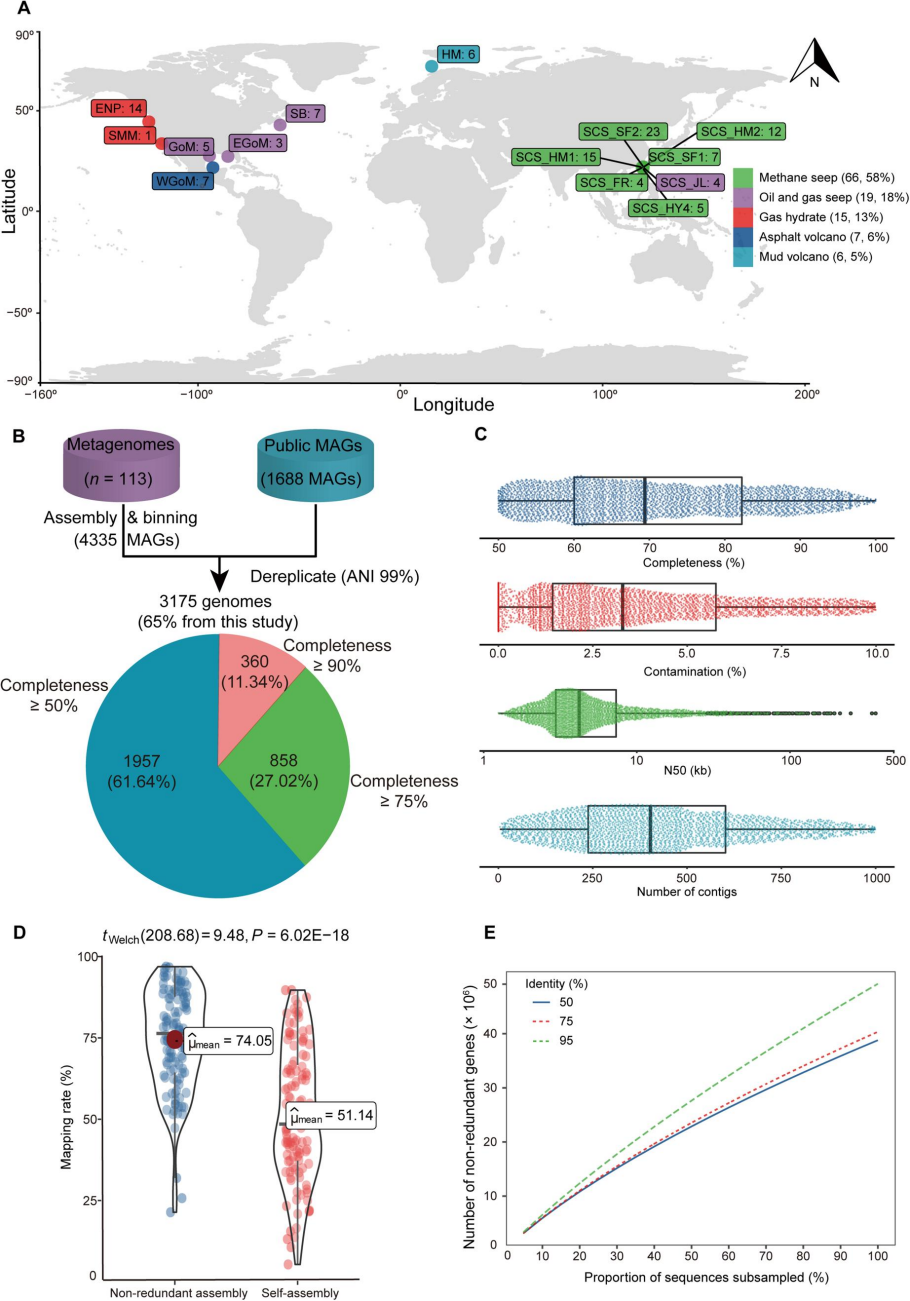
Construction of global CSMD **A**. Geographic distribution of cold seep metagenomes. **B**. Flow-chart on the acquirement of non-redundant cold seep genomes from metagenomes and public MAGs. **C**. Distribution of quality metrics across genomes (*n* = 3175), showing the minimum value, first quartile, median, third quartile, and the maximum value. **D**. Distribution of sample reads mapping rate against 56-Gb non-redundant assembly and self-assembly. Welch’s *t*-test was performed for two groups. **E**. Gene diversity analysis based on 50%, 75%, and 95% nucleotide identity. HM, Haakon Mosby; ENP, Eastern North Pacific; SMM, Santa Monica Mounds; SB, Scotian Basin; GoM, Gulf of Mexico; EGoM, Eastern GoM; WGoM, Western GoM; SCS, South China Sea; JL, Jiaolong; HY4, Haiyang4; SF1, Site F1; SF2, Site F2; FR, Formosa Ridge; HM, Haima; CSMD, Cold Seep Microbiomic Database; MAGs, metagenome-assembled genomes; ANI, average nucleotide identity.

We obtained a total of 113 metagenomic samples from 14 cold seep sites globally, comprising 101 publicly available samples, as well as 12 samples collected by our group. These sites encompassed five distinct types of seepage: methane seep, oil and gas seep, gas hydrate, asphalt volcano, and mud volcano (**Figure 1**A; Table S1). Metagenomic assembly and binning produced 4335 MAGs, which were combined and dereplicated with publicly available 1688 MAGs to finally obtain 3175 non-redundant MAGs (Figure 1B; Table S2). All of them met the medium and above quality level of the Minimum Information about a Metagenome-Assembled Genome (MIMAG) criteria (completeness ≥ 50%, contamination < 10%) [24], with a mean completeness of 71.24% (± 13.45%) and a mean contamination rate of 3.77% (± 2.78%) (Figure 1C). The microbial genomes of cold seep harbor diverse genome sizes (0.50 Mb to 9.26 Mb) and GC contents (23.14% to 72.66%). In addition, 49.87% and 99.94% of total genomes were identified with at least one ribosomal RNA (rRNA) and transfer RNA (tRNA) gene fragments (Table S2).

**Figure 2:**
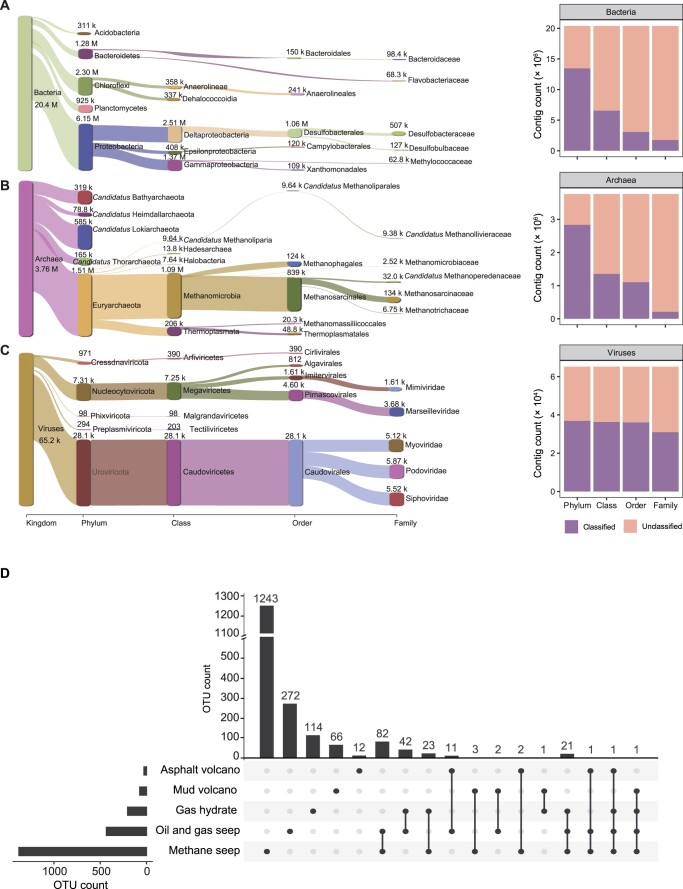
Taxonomic annotation of 56-Gb non-redundant contigs and the distribution of 1895 OTUs across cold seep types **A**.–**C**. Sankey plots based on assigned taxonomy showing the dominant (left) and novel (right) populations of bacteria (A), archaea (B), and viruses (C) at different phylogenetic levels, with the top 5 taxa shown for each level. Numbers on the bar indicate the number of contigs for the lineage (M, × 10^6^; k, × 10^3^). **D**. OTU intersections across sample groups. The UpsetPlot illustrates OTU intersections among cold seep types. OTU, operational taxonomic unit.

Additionally, 113 assembled metagenomes were merged and dereplicated, resulting in a 56-Gb non-redundant contigs of the cold seep microbiome after removing eukaryotic contigs annotated by Contig Annotation Tool (CAT) [25]. A total of 27,599,955 contigs with a mean length of 2.03 kb and a N50 size of 2.08 kb were comprised in this catalog. Among these 56-Gb non-redundant contigs, 73.88%, 13.64%, and 0.24% were taxonomically annotated as bacteria, archaea, and viruses, respectively (**Figure 2**A–C) via CAT annotation [25] based on the National Center of Biotechnology Information (NCBI) Non-Redundant Protein Sequence Database (NR). Proteobacteria, Chloroflexi, Bacteroidetes, Planctomycetes, and Acidobacteria were the top 5 most abundant phyla among bacteria, accounting for 39.74% of total contigs (Figure 2A). Euryarchaeota, *Candidatus* Lokiarchaeota, *Candidatus* Bathyarchaeota, *Candidatus* Thorarchaeota, and *Candidatus* Heimdallarchaeota were the top 5 most abundant phyla among archaea, accounting for 5.49% of total contigs (Figure 2B), while Uroviricota, Nucleocytoviricota, Cressdnaviricota, Preplasmiviricota, and Phixviricota were the top 5 most abundant phyla among viruses, accounting for 0.13% of total contigs (Figure 2C).

The self-mapping analysis showed that a range of 4.92% to 89.23% of reads could be mapped to the respective assembly, with an average mapping rate of 51.14% (± 20.40%) (Figure 1D; Table S3). Nevertheless, when using the 56-Gb non-redundant contigs as the reference, the average mapping rate increased to 74.05% (± 15.45%; ranging from 15% to 96%), representing a 23% improvement in mapping rate on average (Table S3). Therefore, the catalog could be a fundamental reference to facilitate cold seep metagenomic analysis in the future.

Furthermore, a non-redundant protein-coding gene catalog of 49,223,463 gene clusters, representing 71,499,869 full- or partial-length genes, was compiled using an alignment percentage threshold of 80% and a nucleotide identity threshold of 95% [10], with 18.69% of gene clusters containing at least two members. Within the cluster, 33.55% had complete gene representatives based on Prodigal [26] prediction. With the depth of sampling increasing, the number of non-redundant genes increased steadily and did not reach a plateau even at 50% nucleotide identity threshold (Figure 1E). This implies that cold seeps harbor a substantial genetic diversity, necessitating further sequencing efforts to comprehensively capture its functional diversity. Swiss-Prot [27], UniRef50 [28], and NR databases were used to annotate the functions of dereplicated gene clusters, and 33.28%, 79.15%, and 80.26% of genes were hit, respectively. These results suggest that the cold seep microbiome has the potential to encode numerous novel proteins.

### Overview of microbiome composition in cold seeps

By applying an average nucleotide identity (ANI) threshold of 95% in combination with an alignment coverage threshold of 30%, 3175 MAGs were clustered into 1895 operational taxonomic units (OTUs) at species level (Table S2). The 1895 OTUs exhibited low sequence identities with genomes from other environmental bacterial and archaeal genomic databases according to the threshold of 95% ANI, including the Tibetan Glacier Genome and Gene (TG2G) (100% novelty) [10], the TARA Oceans genomes (100% novelty) [20], the Ocean Microbiomics Database (OMD) (99.21% novelty) [11], the Genomes from Earth’s Microbiomes (GEM) (97.79% novelty) [13], and the Genome Taxonomy Database (GTDB) R06-RS202 (94.41% novelty) [29] (Table S4). Approximately 90% (1707 OTUs, 89.98%) of the OTUs were present in only one cold seep type, 8.75% (166 OTUs) in two types, and only 1.27% (24 OTUs) in three or more types (Figure 2D; Table S5). Similarly, the abundance of OTUs showed a high degree of niche specialization of cold seep (Figure S1; Table S6). Thus, further investigation of cold seep microbial diversity is necessary.

According to the GTDB (release R06-RS202) [30] annotation, the cold seep genomic dataset showed a substantial taxonomic diversity. The 1895 OTUs spanned across 105 phyla, 173 classes, 308 orders, 433 families, and 407 genera (Table S2). In addition, the numbers of species in 17 under-represented phyla were expanded for 1.25–4 times compared to the GTDB R06-RS202 [29] (Table S7). For example, uncultured UBP7_A increased by 3-fold, Krumholzibacteriota increased by 1.1-fold, and Asgardarchaeota increased by 0.28-fold (Table S7). Furthermore, 46 classes, 130 orders, 297 families, 960 genera, and 1790 species represented potential novel lineages compared to the GTDB. Chloroflexota (200 OTUs, 10.06%), Proteobacteria (197 OTUs, 9.91%), Desulfobacterota (145 OTUs, 7.29%), Planctomycetota (105 OTUs, 5.33%), and Patescibacteria (105 OTUs, 5.33%) were the five phyla of bacteria that contained a relatively high number of species, while Halobacteriota (75 OTUs, 3.77%), Asgardarchaeota (74 OTUs, 3.72%), Thermoplasmatota (68 OTUs, 3.42%), Thermoproteota (52 OTUs, 2.62%), and Nanoarchaeota (40 OTUs, 2.01%) were the phyla of archaea with a relatively high number of species (Table S2). Even with 296 high-quality OTUs (completeness > 90%), 10 classes, 21 orders, 38 families, 124 genera, and 254 species represented potential novel lineages (Table S2).

The fluid systems of cold seeps are usually classified as mineral-prone systems (*e.g*., methane seep, oil and gas seep, and gas hydrates) with low discharge and mud-prone systems (*e.g*., mud volcano and asphalt volcano) with high discharge, according to the fluid flow regime [1]. We investigated the microbial composition across sampling sites and cold seep types based on the relative abundance of OTUs. In terms of the average relative abundance, Halobacteriota (18.74%), Desulfobacterota (15.2%), Chloroflexota (12.07%), Caldatribacteriota (9.47%), and Proteobacteria (8.48%) represented as the most abundant phyla (Figure S2A). Simpson’s and Shannon’s diversity of the cold seep microbiomes were significantly higher (*P* < 0.01) in mineral-prone systems than in mud-prone systems based on OTUs (Figures S2B and S3). Meanwhile, principal co-ordinates analysis (PCoA) of microbial communities using Bray–Curtis distance showed that sampling sites had a greater impact on the distribution of microbiome communities compared to cold seep types at the phylum level of MAGs (Figure S2C) and 16S (miTags) (Figure S4; Table S8).

### Versatile metabolic potential of the CSMD

**Figure 3:**
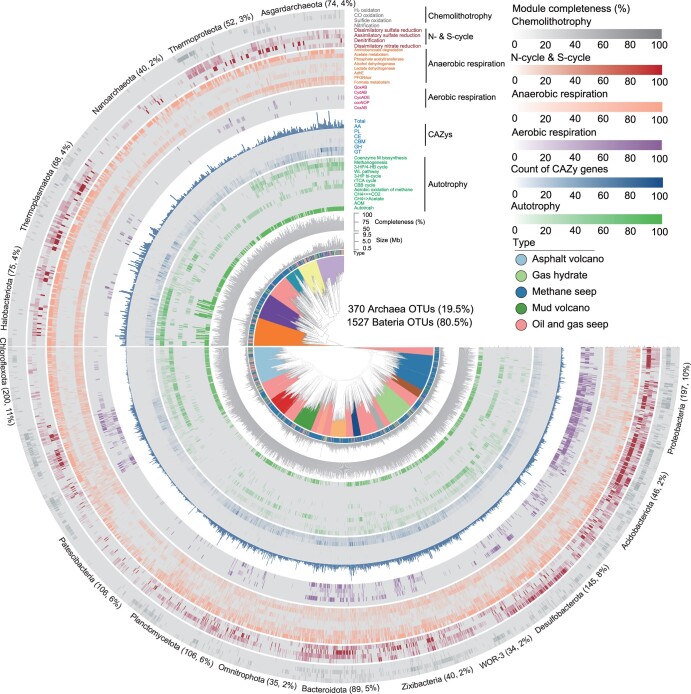
Phylogenetic distribution and metabolic profile of 1895 OTUs in the CSMD The phylogenetic tree was inferred using IQ-TREE from an aligned concatenated set of 120 single-copy marker proteins for bacteria, and from a concatenated set of 122 marker proteins for archaea. The key genes of the following pathways are displayed in the diagram: AOM; CAZys, including GH, GT, PL, CE, AA, and CBM; CO_2_ fixation, including the WL pathway, CBB cycle, rTCA cycle, 3-HP bi-cycle, and 3-HP/4-HB cycle; anaerobic respiration; aerobic respiration; and chemolithotrophy (refer to Table S9 for details). AOM, anaerobic oxidation of methane; CAZy, carbohydrate-active enzyme; GH, glycosidase or glycosyl hydrolase; GT, glycosyltransferase; PL, polysaccharide lyase; CE, carbohydrate esterase; AA, auxiliary activity; CBM, carbohydrate-binding module; WL, Wood–Ljungdahl; CBB, Calvin-Benson-Bessham; rTCA, reverse tricarboxylic acid; 3-HP, 3-hydroxypropionate; 3-HP/4-HB, 3-hydroxypropionate/4-hydroxybuty.

To study the metabolic potential of the cold seep microbiome, 1895 OTUs were functionally annotated based on the Kyoto Encyclopedia of Genes and Genomes (KEGG) database. We first investigated the anaerobic oxidation of methane (AOM) pathway, a metabolic process that is a primitive driver of the cold seep ecosystem. We found that 30 OTUs contained the marker genes for the AOM pathway. Among them, 13 had the complete genes involved in the oxidation of methane to CO_2_, 29 had the complete genes from methane to acetate, and 12 had both metabolic steps (**Figure 3**; Table S9). All these 30 OTUs were affiliated to ANME, with 22 of them representing novel genera or species. Additionally, we found that 1163 OTUs (61.31%; 90 phyla) contained at least one of five pathways for CO_2_ fixation (Figure 3; Table S9). Among them, the Wood–Ljungdahl (WL) pathway (636 OTUs) was the most prevalent, followed by the Calvin–Benson–Bessham (CBB) cycle (402 OTUs), the 3-hydroxypropionate-4-hydroxybutyric acid (3-HP/4-HB) cycle (240 OTUs), the 3-hydroxypropionate (3-HP) bi-cycle (170 OTUs), and the reverse tricarboxylic acid (rTCA) cycle (107 OTUs) (Figure 3; Table S9). The top 5 most widely distributed phyla harboring the WL pathway were Chloroflexota, Desulfobacterota, Planctomycetota, Thermoplasmatota, and Halobacteriota. The WL pathway in bacteria has been widely discovered, with experimental validation or computational inference in Chloroflexota [31] and Desulfobacterota [32] in the ocean. Compared to that in other organisms, the WL pathway in archaea is poorly understood [33]. A recent study has shown that Thermoplasmatota has the ability to perform autotrophic growth via the WL pathway [34], and we identified 27 OTUs belonging to Thermoplasmatota with this pathway. Interestingly, we also found 22 OTUs from Halobacteriota possessing key enzymes for the WL pathway, which has not been reported before. Further experiments are required to confirm this *in silico* observation. To explore the heterotrophic potential of the cold seep microbiome, we investigated the genes involved in carbohydrate degradation and found that 1887 OTUs might perform heterotrophic metabolism (Figure 3; Table S9). A novel species from Planctomycetota (SRR13892593_me2_bin.111) possessed the most numerous genes (145 genes) for carbohydrate degradation. Totally, 1163 OTUs (61.31%) belonging to 90 phyla that encode both the carbohydrate-degrading enzymes and any of the inorganic carbon fixation pathways were considered as potential mixotrophs [35], albeit not rigorously so.

Oxygen requirement analysis revealed that all OTUs had at least one anaerobic respiratory pathway. As an illustration, our analysis found the presence of 1296 OTUs with formate metabolism, 582 OTUs with lactate dehydrogenase, 752 OTUs with alcohol dehydrogenase, 1583 OTUs with acetate metabolism, and 1116 OTUs with aminobenzoate degradation (Figure 3; Table S9). Furthermore, we investigated the potential of the cold seep microbiome to perform aerobic respiration. In total, 736 OTUs (38.79%) were found to contain aerobic respiration genes, such as cytochrome c oxidase (Cox/Cyd/Qox/Cco/Cyo) genes (Figure 3; Table S9). These OTUs were associated with 44 phyla, such as Proteobacteria, Asgardarchaeota, Halobacteriota, Chloroflexota, and Nanoarchaeota. All 736 OTUs might perform aerobic respiration using at least one of the anaerobic respiration pathways, indicating potential facultative anaerobic capabilities. These species spanned 55 phyla, primarily including Proteobacteria (181 OTUs), Chloroflexota (101 OTUs), Bacteroidota (78 OTUs), and Desulfobacterota (54 OTUs). Taking together, cold seep microorganisms are prevalent for anaerobic respiration, while also accompanied by substantial genes involved in aerobic respiration.

### Biosynthetic potential of the CSMD

**Figure 4:**
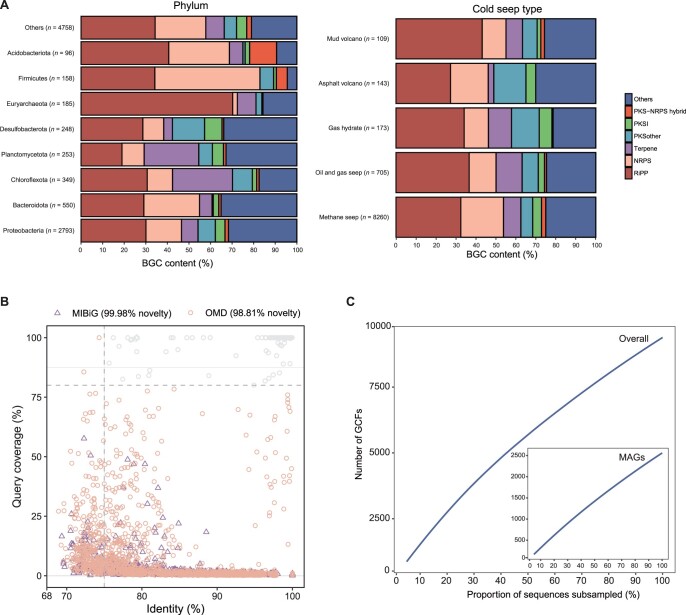
The diversity and novelty of BGCs identified in cold seep microbiomes **A**. The relative frequency of BGC classes across dominant phyla (left) and cold seep types (right). **B**. Comparing GCFs to experimentally validated MIBiG and computationally predicted OMD BGCs uncovers the novelty of GCFs. Only results with BLASTN E-value less than 1E−5 were shown. **C**. Rarefaction curves of GCFs derived from all contigs and MAGs. BGC, biosynthetic gene cluster; MIBiG, Minimum Information about a Biosynthetic Gene; OMD, Ocean Microbiomics Database; GCF, gene cluster family; NRPS, non-ribosomal peptide synthase; PKS, polyketide synthase; RiPP, ribosomally synthesized and post-translationally modified peptide.

To explore the value of the CSMD, we analyzed its potential in the assessment of natural product synthesis. We identified 17,968 putative BGCs with an average length of 7.85 kb (± 6.96 kb) from the cold seep assemblies using antiSMASH (v5.1) [36] (Table S10). To reduce the effect of incomplete and redundant BGCs, these were clustered into 9390 gene cluster families (GCFs) with an average length of 8.54 kb (± 7.63 kb). This was nearly 3.75 times the number of function-known BGCs within Minimum Information about a Biosynthetic Gene (MIBiG) (https://mibig.secondarymetabolites.org/stats) [37], demonstrating the high diversity of BGCs in the cold seep microbiome. We found that 29.61% of the GCFs had two or more members (Table S10). Collectively, 3112 (33.14%) GCFs containing NRPSs and PKSs were identified from 70 phyla (Figure S5; Table S10), 3082 (32.82%) GCFs containing RiPPs were identified from 17 phyla, and 845 (8.99%) GCFs containing terpenoids were identified from 10 phyla, with the above accounting for 75% of the total (Figure S5; Table S10). This may be due to the wide involvement of RiPP-like, ranthipeptide, and thiopeptide BGCs in quorum sensing, osmotic stress, and the regulation of cellular metabolism in cold seep microorganisms [11,38,39]. It is noteworthy that BGC types showed substantial variation among different phyla, but their distribution across cold seep types appeared to be relatively consistent (**Figure 4**A). This could be attributed to the fact that different phyla commonly carry genes that encode particular natural products. For instance, Chloroflexota and Planctomycetota frequently possess genes involved in terpene synthesis [10,13], whereas Firmicutes typically harbor genes associated with NRPS synthesis [11,13].

To assess the novelty of the BGCs identified in this study, we compared representative GCFs to MIBiG and OMD. By using BLASTN with a threshold of 80% query coverage and 75% identity [40], only two GCFs were identified in MIBiG, while 98.81% (9278) of the GCFs were considered as novel BGCs compared to OMD (Figure 4B), suggesting the potential for encoding novel chemical components. For example, one PKS–NRPS hybrid cluster of 84,733 bp comprising ten core modules, identified from a MAG (SRR13892603_vb_S1C4173) classified as a novel genus in family UBA2199 (Figure S5A), showed the highest similarity [71% amino acid identity (AAI)] to the antibiotic sevadicin biosynthesis gene cluster of *Paenibacillus larvae*. Likewise, a RiPP cluster of 44,319 bp with four core modules, identified from a MAG (SRR13892601_vb_S1C33830) classified as a novel species of Omnitrophota (Figure S5B), exhibited the highest similarity (28% AAI) to the antibiotic ranthipeptide of *Streptococcus mutans* UA159. In addition, as the sampling BGCs increased, the number of GCFs steadily increased, whether originating from MAGs or contigs (Figure 4C), suggesting that BGCs in cold seeps warrant further exploration to be in line with the trend of taxonomic exploration.

### Phylogenetic distribution of BGC-rich clades

**Figure 5:**
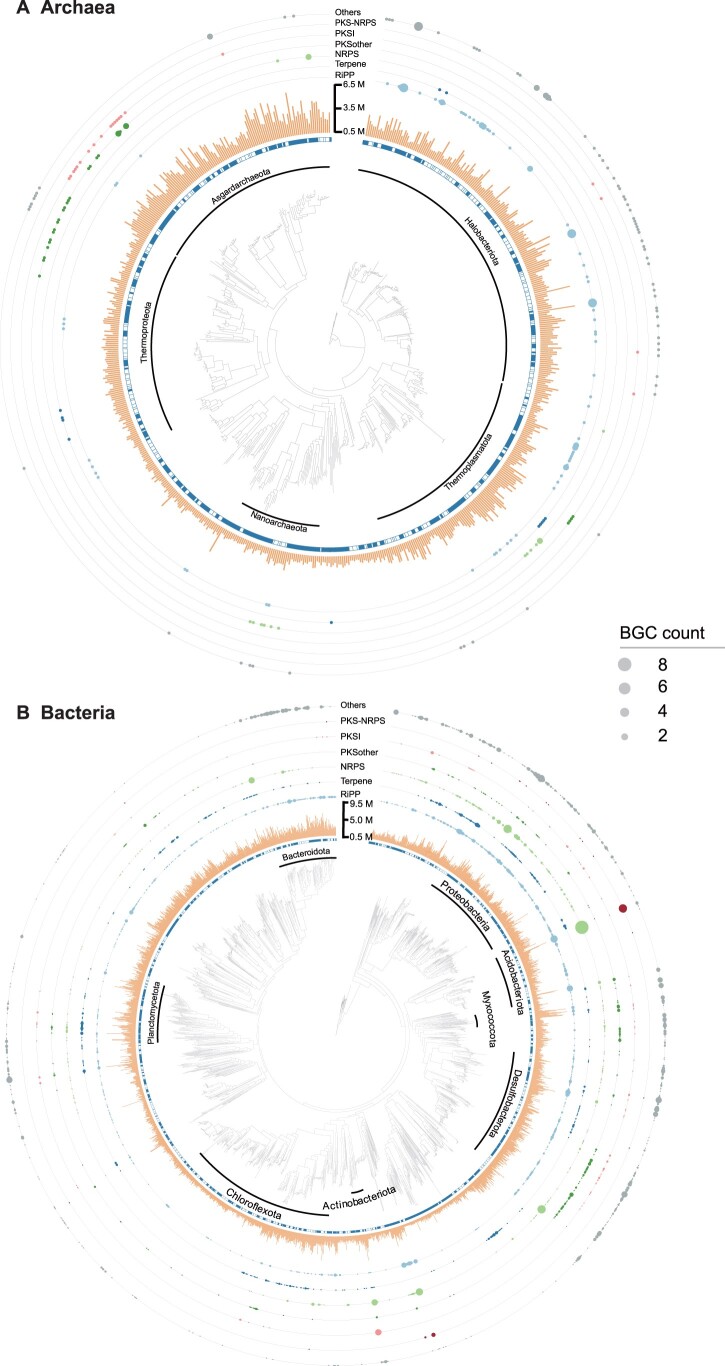
Illustration of BGC-rich lineages in cold seep microbiomes The solid square in the innermost circle indicates the representative genome of each OTU. The circle size indicates the number of BGCs for each category. **A**. Archaea. **B**. Bacteria.

To better reveal the relationship between cold seep microbial taxonomy and natural product synthesis, we mapped the phylogenetic distribution of BGC-rich clades. For this purpose, 3175 MAGs were placed in standardized bacterial and archaeal phylogenetic trees of GTDB and the numbers of BGC types were overlaid (**Figure 5**A and B; Table S11). In total, 45.92% (1458) of MAGs contained at least one BGC, with an average length of 9.8 kb (± 8.8 kb). Notably, bacteria had a higher BGC count per genome than archaea (bacteria *vs.* archaea: 2.38 ± 1.94 *vs.* 1.28 ± 0.62; *P* < 0.001, Mann–Whitney test) (Table S11). After normalizing for genome size, bacteria displayed an even higher BGC count per Mb compared to archaea (bacteria *vs.* archaea: 1.01 ± 0.70 *vs.* 0.78 ± 0.39; *P* < 0.00001, Mann–Whitney test) (Table S10). The results indicate that bacteria exhibit a greater potential for natural product biosynthesis than archaea. MAGs for Proteobacteria, Desulfobacterota, Bacteroidota, Chloroflexota, and Planctomycetota are the bacterial phyla with the highest number of BGCs, consistent with the predictions based on all contigs (Figure 4A). In addition, 238 BGCs were detected within Halobacteriota (110 BGCs), Thermoplasmatota (45 BGCs), Asgardarchaeota (36 BGCs), Thermoproteota (34 BGCs), and Nanoarchaeota (13 BGCs), with a predominance of RiPPS, NRPS, and PKS groups (Table S11). Overall, the CSMD provides access to novel lineages, offering microbial resources for the discovery of novel natural products.

**Figure 6:**
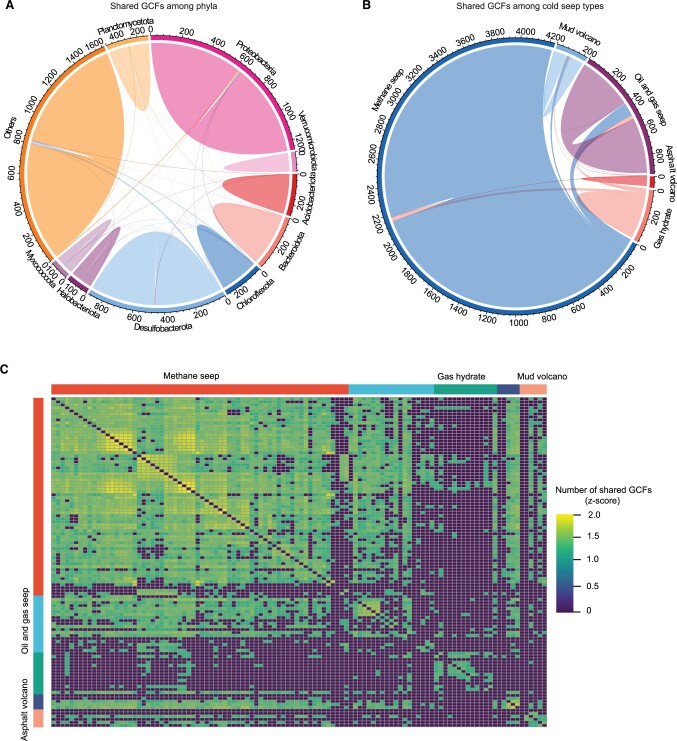
The distribution of GCFs among phyla and cold seep types **A**. Shared GCFs among phyla (solid shapes), with pairwise overlaps among phyla (ribbons). **B**. Shared GCFs among cold seep types, with pairwise overlaps among cold seep types (ribbons). **C**. Log_10_-normalized pairwise heatmap of shared GCF counts among samples.

Afterward, to investigate the overlap of the natural product synthesis potential among different phyla and cold seep types, we examined the distribution of GCFs within each phylum and cold seep type (**Figure 6**A and B). In most phyla, the majority (73.81% ± 20.35%) of GCFs appeared to be phylum-unique (Figure 6A). Likewise, shared GCFs were rarely observed among cold seep types, with most detected in only one type (Figure 6B). Exceptionally, a few shared GCFs were observed between methane seep and oil/gas seep in MAGs (Figure 6B) and samples (Figure 6C). This may be due to that these two types share similar environmental factors [1,2].

## Discussion

Although prior investigations [2,15,41–44] have focused on cold seep communities and metabolic research, a comprehensive metagenomic-based dataset on a global scale remains lacking. Here, we present a specialized and fully integrated microbiome genome and gene catalog for the global cold seep ecosystem. Compared to the previously published 1688 MAGs [15,16,19,43,44], the CSMD incorporates a 65% increase in genomes at the 99% ANI level, including 33 new phyla, 105 new classes, 247 new orders, 360 new families, 380 new genera, and 1094 new species (Table S12). Apart from MAGs, we also acquired unbinned contigs and integrated them with all MAGs to create 56-Gb non-redundant contigs, an aspect that has been neglected in past investigations [19,44]. This dataset is expected to be a fundamental resource for further exploration of evolution and gene function like glacier [10], marine [11], and human gut [12] databases.

We observed that Halobacteriota and Desulfobacterota were the dominant phyla in terms of relative abundance in cold seeps globally, which is not surprising in line with their inclusion of typical ANME/SRB consortia in cold seeps [45]. Interestingly, a high abundance of Caldatribacteria was exclusively distributed in the gas hydrate type, consistent with a previous 16S-based study [5]. A recent study on species within Caldatribacteria isolated from gas hydrates indicates that environmental adaptation may be linked to its cell membrane structure [46]. However, whether Caldatribacteria dominates in gas hydrate type remains to be explored [5]. In addition, we found that mineral-prone systems exhibit higher alpha diversity than mud-prone systems, which is consistent with previous studies focusing on viral communities [15]. This may be attributed to the longer geological history and slower fluid discharge of mineral-prone systems, providing a more stable living environment for microorganisms compared to young and fast mud-prone systems [1]. Additionally, studies based on 16S sequencing have shown that both sampling site and cold seep type significantly affect microbial community composition [47], as confirmed by our results as well. Our findings also indicate that the sampling site exerts a stronger effect than the cold seep type, which is not a surprise considering the strong influence of environmental heterogeneity on microorganisms (small-scale spatial variation, even in the centimeter or micrometer range, may lead to dramatic changes in nutrient conditions) [48].

We discovered a rich repertoire of metabolic pathways in the cold seeps. Firstly, we found that the WL pathway was the most common carbon fixation pathway among cold seep microorganisms. Compared to the CBB and rTCA cycles, the WL pathway exhibits lower ATP demand, higher efficiency, and a faster rate [49], making it possibly a more economical choice for cold seep microorganisms. Secondly, we identified that 90% of the OTUs might have the potential to degrade organic compounds based on the genes involved in carbohydrate degradation [35]. The organic compounds, including carbohydrates, are produced by AOM and settle from the upper layers of the ocean, providing a substantial nutritional status for the cold seep microbiome [1,45]. Additionally, compared to the 39% (69 out of 178 MAGs) mixotrophic ratio of microorganisms in the Challenger Deep [35], the proportion in cold seeps reaches 61%, which may be due to the richer availability of inorganic and organic carbon sources in cold seeps [1]. Although the strict definition of mixotrophic ability is complex and usually requires experimental verification using microbial isolates, our results can be regarded as a preliminary, albeit rough, exploration.

With the limited availability of oxygen in a few millimeters to centimeters of the sediment surface, cold seep sediments are typically hypoxic [1]. As expected, we observed that almost all OTUs contained at least one anaerobic metabolism pathway, indicating the dominance of anaerobic metabolism in cold seeps, consistent with previous reports based on experimental and computational approaches [6,50]. Interestingly, we also found that up to 39% of OTUs exhibited potential facultative anaerobic respiratory capabilities. Similar results have been reported in studies of the Challenger Deep [35], possibly due to the high pressure, absence of light, and low oxygen in both environments. Although more experiments are needed, these results suggest that the facultative anaerobic respiratory capabilities in cold seep microbiome may have been underestimated.

The discovery of highly novel BGCs has also been evident in microbiomes from marine [11] and glacier [10] environments, indicating a widespread potential for environmental microorganisms to synthesize novel natural products, which is consistent with the high novelty of environmental microbial genomes. Given that the majority of currently identified natural products primarily derive from a limited number of cultivable microbial groups [37], the high novelty of BGCs in environments such as cold seeps, which harbor a large proportion of uncultivated microorganisms, is not surprising. Interestingly, we found that Desulfobacterota possessed considerable biosynthetic potential, a trait also observed in microorganisms from the permanently anoxic Cariaco Basin [51], suggesting that Desulfobacterota may represent a lineage with unique biosynthetic encoding potential in anoxic environments. The biosynthetic potential of archaea has recently gained attention [51], and our findings indicate that over 27% of archaea MAGs encode BGCs. We anticipate that the archaea could provide an even more extensive potential for novel natural products.

In summary, the CSMD serves as a valuable repository and platform for archiving, analyzing, and contrasting cold seep microbiomes at the genomic and genetic levels. Here, we demonstrate its distinctive utility in exploring microbial taxonomic and functional diversity. This comprehensive work not only fills the knowledge gap in comprehending microbial diversity and function within global cold seep ecosystem, but also provides a rich resource for natural product bioprospecting. We expect that the catalog will facilitate the research on the global cold seep microbiome as additional studies become accessible.

## Materials and methods

### Metagenomic sample collection

Overall, 113 metagenomic samples, including both proprietary and publicly available data, were collected from different sites around the world, covering 5 different cold seep types (Figure 1A; Table S1). Among them, the SCS_HM2 dataset with 12 samples was obtained from the active Haima cold seep of the South China Sea (22°07′ N, 119°17′ E) (Table S1) at a water depth of 1100 m during research expeditions conducted by the scientific cruise Research Vessel “KEXUE” in 2017. The Haima seeps are characterized by abundant carbonate rocks and are inhabited by a large number of living and dead bivalves [52]. Among the samples, seven (HTR2, HTR3, HTR4, HTR5, HTR7, HTR11, and HTR12) were collected by grab sampler from the sediment surface [approximately 0–0.02 m below seafloor (mbsf)], while three samples (HTR8, HTR9, and HTR10) were collected by remotely operated vehicle (ROV) push cores from soil depths of approximately 0.02–0.2 mbsf. The remaining two samples (HTR1 and HTR6) were collected by a gravity corer from soil depths of 0–1.6 mbsf. The uppermost layer impacted by seawater was discarded, and the sediment located at the core of each section was collected and stored in anaerobic biobags at −80°C for future utilization. The data from the remaining 101 samples were downloaded from NCBI’s Sequence Read Archive (SRA) (Table S1) [4–7,14,16–19,41–43].

### DNA extraction and metagenomic sequencing of SCS_HM2 samples

The total DNA from SCS_HM2 sediments (∼ 0.5 g) was extracted using the PowerSoil DNA Isolation Kit (Catalog No. 12888-50, Qiagen, Germantown, MD) following the manufacturer’s instructions. Genomic libraries were constructed and sequenced on the Beijing Genomics Institute (BGI) MGISEQ-2000RS platform at the National Microbiology Data Center, Institute of Microbiology, Chinese Academy of Sciences (Beijing, China) with a 150 bp paired-end model, followed by standard data processing protocols.

### Metagenomic quality control and assembly

The quality control of raw reads was performed via Trim Galore (v0.5.0; https://www.bioinformatics.babraham.ac.uk/projects/trim_galore/). Only paired reads with sequence length ≥ 100 bp were retained after adapter sequences were removed, and low-quality reads were trimmed from the 3′-primer end with a Phred quality score (*Q*) threshold of 30. The 113 metagenomes were assembled with MEGAHIT (v1.1.3) [53] using the default *k*-mer parameters (--k-list 21, 29, 39, 59, 79, 99, 119, 141), retaining contigs greater than 1000 bp in length. The overall mapping rate of each sample was calculated by Bowtie 2 (v2.3.5) [54] with default parameters.

### Construction of non-redundant genome and contig taxonomic annotation

The contigs derived from 113 metagenomes and public MAGs with lengths over 1 kb were dereplicated at 90% aligned region and 95% nucleotide identity using MMseqs2 [55] with the parameters “easy-linclust -e 0.001, -min-seq-id 0.95 and -c 0.9” [56]. Subsequently, all contigs were taxonomically annotated by CAT (v5.2.3) [25] with default parameters based on the NCBI NR database (v2021-01-07). The 56-Gb non-redundant contigs of the cold seep microbiome were obtained after removing eukaryotic sequences (mainly *Mytilus galloprovincialis* and *Pomacea canaliculata* that commonly accompany the Mollusca phylum in cold seeps).

### Metagenome binning and genome quality control

Metagenomic assemblies were binned using MetaBAT2 (v2.12.1) [57], MaxBin2 (v2.2.7) [58], and CONCOCT [59] wrapped in MetaWRAP (v1.3.2) [60] with default parameters for each sample. In addition, VAMB (v2.0.1) [61] was also used for binning based on deep variational autoencoders. Subsequently, the completeness and contamination of bins were calculated using the “lineage_wf” module of CheckM (v1.0.12) [62]. tRNA genes were identified using ARAGORN [63], and rRNA genes were identified using Barrnap (v0.9; https://github.com/tseemann/barrnap). Finally, 4335 MAGs meeting the medium and above quality of MIMAG [24] were retained for subsequent analysis.

### Genome dereplication and generation of species-level OTUs

A total of 3246 previously public MAGs [15,16,19,43,44] were collected and dereplicated to 1688 genomes by dRep (v3.2.0) [64] based on genome-wide ANI percentage threshold of 99% with the following parameters: -comp 50, -con 10 and -sa 0.99. Subsequently, 3175 non-redundant genomes were obtained by dRep with the ANI threshold of 99% combined with the previous 4335 MAGs. Finally, 1895 representative species-level OTUs were clustered using dRep based on the aligned coverage of over 30% and the ANI threshold of 95% (-nc 0.3, -sa 0.95) [10,13].

### MAG abundance, alpha diversity, and beta diversity analyses

Quality-controlled reads were mapped to MAGs using minimap2 [65] with default parameters. The abundance of MAGs was calculated using CoverM (v0.6.0; https://github.com/wwood/CoverM) with parameters: --min-read-aligned-percent 0.75, --min-read-percent-identity 0.95, --proper-pairs-only, --methods tpm. Transcripts per million (TPM) was used to eliminate the effects of sample sequencing depth and genome length [10,66]. In addition, phyloFlash (v3.4.1) [67] was used for extracting 16S miTags from clean metagenomic data by parameters “-almosteverything”, followed by classifying via SILVA database (v138.1) [68]. Subsequently, the “rarecurve” function in the vegan package (https://github.com/vegandevs/vegan/) of R was used to assess sample sequencing saturation and remove samples with low sequencing depth. Taxonomic structure plot, alpha diversity, and beta diversity analyses were performed using the R package EasyMicroPlot [69]. Mann–Whitney test was used for two groups of Shannon as well as Simpson indices, and one-way analysis of variance (ANOVA) and Tukey Honestly Significant Difference (HSD) post-hoc tests were applied among groups [70]. For beta diversity analysis, Bray–Curtis distances were measured, and PERMANOVA analysis was used to test for statistical significance among different independent variables with the default settings (999 permutations).

### Comparison of MAGs and genomes of public databases

The species-level representative OTUs were compared to 103,722 publicly available reference genomes, including 968 genomes from the TG2G [10], 957 MAGs from the TARA Oceans [20], 8304 MAGs from the OMD [11], 45,599 MAGs from the GEM [13], and 47,894 MAGs from the GTDB [29]. Each reference dataset was compared with 1895 OTUs using dRep. A cold seep OTU was designated as a novel species, which exhibited an ANI of less than 95% when compared with other reference genomes [10].

### Metabolic pathway analysis of MAGs

Genes were predicted for MAGs using Prokka (v1.14.6) [71] with single genome model. The KEGG pathway was then annotated by eggNOG-mapper (v2.1.6) [72] based on eggNOG database (v5.0) [73]. To elucidate an overview of the specific metabolic modules of each MAG, the key enzymes of the metabolic pathway were summarized and visualized following the method of Chen and his colleagues [35]. The module completeness of a given metabolic pathway was quantified as the percentage of identified key marker genes present in the corresponding pathway. For example, a module completeness value of 50 indicates that the MAG contains 50% of the marker genes in the complete pathway [35].

### Taxonomic annotation and phylogenetic tree inference

The taxonomic annotation of the 3175 MAGs was performed using the Genome Taxonomy Database Toolkit (GTDB-Tk; v0.3.2) [30] with the GTDB database release R06-RS202 [47]. MAGs were classified at the species level if the ANI to the closest GTDB-Tk representative genome was ≥ 95% and the aligned coverage was ≥ 60%. Finally, the phylogenetic tree was inferred by IQ-TREE (v2.2.0-beta) [74] with parameters: -B 1000, -m LG + G, -wbtl, based on the concatenated multiple sequence alignments of 122 archaeal or 120 bacterial universal marker genes generated by GTDB-Tk after trimming sequence gaps via trimAl (v1.4.rev15) [75]. iTOL [76] was used to visualize phylogenetic trees.

### Gene function annotation

The prediction of open reading frames (ORFs) in metagenomic assemblies was carried out using Prodigal [26]. The resulting ORFs were then dereplicated by clustering at 80% aligned region with 95% nucleotide identity, employing MMseqs2 [50] with the parameters: easy-linclust -e 0.001, --min-seq-id 0.95, -c 0.80 [10]. The gene rarefaction analysis was performed using an in-house Python script, based on the gene cluster results of MMseqs2 with identity thresholds of 95%, 75%, and 50% (easy-linclust -e 0.001 -c 0.80) [10]. The analysis was repeated 100 times with a 5% sampling step. Further, the function of the non-redundant gene catalog was annotated to the Swiss-Prot [27], UniRef50 [28], and NR databases via MMseqs2 [55] with parameters: easy-search -e 0.01, --min-seq-id 0.3, --cov-mode 2 -c 0.8.

### The secondary metabolite BGC analysis

The secondary metabolite BGC was predicted for contigs ≥ 3 kb in length using antiSMASH (v5.1) [36] with default settings. Subsequently, the BGCs were categorized into GCFs and labeled with seven categories: “PKSI”, “PKS–NRPS hybrid”, “PKSother”, “NRPS”, “RiPP”, “Terpene”, and “Others”, based on the results of the BiG-SCAPE [21] with default parameters.

### Novelty of GCFs

The novelty of GCFs was estimated based on the result of BLASTN (BLAST 2.2.28+) [40,77] to databases of experimentally validated MIBiG 2.0 [37] and the latest computationally predicted OMD [11]. For the representative BGC of GCFs, we selected the sequence with maximum query coverage and identity to the respective database as the best hit. A GCF was deemed novel if the best hit to the reference was below 80% query coverage and 75% identity following the threshold of GEM [13].

## Supplementary Material

qzad006_Supplementary_Data

## Data Availability

The raw reads of 12 samples in this study have been deposited in the NCBI Sequence Read Archive (BioProject: PRJNA916811) which are publicly accessible at https://www.ncbi.nlm.nih.gov/sra, the National Microbiology Data Center (NMDC: NMDC10018281) which are publicly accessible at https://nmdc.cn/, and the Genome Sequence Archive [78]  at the National Genomics Data Center (NGDC), Beijing Institute of Genomics (BIG), Chinese Academy of Sciences (CAS) / China National Center for Bioinformation (CNCB) (GSA: CRA010074) which are publicly accessible at https://ngdc.cncb.ac.cn/gsa. The genome sequences of 3175 MAGs and 54-Gb non-redundant contigs of CSMD have been deposited in the Genome Warehouse [79] at the NGDC, BIG, CAS / CNCB (BioProject: PRJCA015385) which are publicly accessible at https://ngdc.cncb.ac.cn/gwh, and also available from https://doi.org/10.6084/m9.figshare.21731330.

## CRediT author statement

**Tao Yu:** Conceptualization, Methodology, Investigation, Formal analysis, Writing – original draft. **Yingfeng Luo:** Conceptualization, Writing – review & editing. **Xinyu Tan:** Methodology. **Dahe Zhao:** Methodology. **Xiaochun Bi:** Methodology. **Chenji Li:** Methodology. **Yanning Zheng:** Methodology, Writing – review & editing. **Hua Xiang:** Conceptualization, Writing – review & editing. **Songnian Hu:** Conceptualization, Writing – review & editing, Supervision. All authors have read and approved the final manuscript.
