# Supplementary material for: Global Marine Cold Seep Metagenomes Reveal Diversity of Taxonomy, Metabolic Function, and Natural Products
Source: Genomics Proteomics Bioinformatics. 2023 Dec 13;22(2):qzad006. doi: 10.1093/gpbjnl/qzad006 (PMC12016038; doi:10.1093/gpbjnl/qzad006)
Supplement: qzad006_Supplementary_Data [file qzad006_supplementary_data.zip › Supplementary material captions.docx]

# Supplementary material

**Figure S1 Heatmap presentation of relative abundance of CSMD 1895 OTUs among samples**

The abundance of 1895 OTUs in 113 samples was normalized by z-score and bi-directionally clustered. Cold seep types are shown as column annotations, and OTUs are shown as row annotations. TPM, transcripts per million.

Figure S2 Cold seep microbiome composition barplot, alpha diversity, and beta diversity based on MAGs abundance at phylum level

**A.** Taxonomic relative abundance barplot of cold seep microbiome. **B.** Simpson and Shannon diversity between mineral-prone system and mud-prone system. Statistics by Mann–Whitney test, with ** indicating the significance at *P* < 0.01. **C.** Beta diversity difference among cold seep sites and types based on Bray–Curtis dissimilarities. Ellipses represent 95% confidence contours of samples grouped by cold seep type. PERMANOVA analysis was used to test for statistical significance for the main effects of cold seep sites and types. PCoA, principal co-ordinates analysis.

Figure S3 Simpson and Shannon diversity among cold seep types

Different letters on the bar indicate the values that differ significantly among cold seep types at *P* < 0.05 (one-way ANOVA and Tukey HSD post-hoc tests). ANOVA, analysis of variance; HSD, Honestly Significant Difference.

Figure S4 Cold seep microbiome beta diversity and heatmap based on 16S abundance at phylum level

**A.** Beta diversity difference among cold seep sites and types based on Bray–Curtis dissimilarities. Ellipses represent 95% confidence contours of samples grouped by cold seep type. PERMANOVA analysis was used to perform statistical significance for the main effects of cold seep sites and types. **B.** The heatmap of 16S abundance normalized by z-score and clustered bi-directionally. Cold seep types are shown as column annotations, and OTUs are shown as row annotations.

Figure S5 The genomic structures of two identified BGCs

BGCs are predicted from a novel genus (SRR13892603_vb_S1C4173) and a species (SRR13892601_vb_S1C33830) encoding a PKS–NRPS hybrid and a RiPP gene cluster, respectively. A. The PKS–NRPS hybrid cluster of SRR13892603_vb_S1C4173 comprises 10 core modules and spans 84,733 bp. B. The RiPP cluster of SRR13892601_vb_S1C33830 comprises 4 core modules and spans 44,319 bp.

**Table S1 The global cold seep metagenome and assembly statistics**

**Table S2 The genomic characteristics of 3175 MAGs**

**Table S3 The read mapping rate of self-assembly and 56 Gb non-redundant contigs**

**Table S4 The novelty comparison of CSMD OTUs with public genomes**

**Table S5 The shared OTU members among cold seep types**

**Table S6 The OTU abundance among samples**

**Table S7 Taxonomic expansion of CSMD compared with GTDB** **R06-RS202 at the phylum level**

**Table S8 Reads count of 16S (miTags) among samples**

**Table S9 The metabolic profile of 1895 OTUs**

**Table S10 A summary of 17,968 BGCs**

**Table S11 The BGC class count of MAGs**

**Table S12 Taxonomic expansion of CSMD compared to public MAGs of cold seeps**
