# Supplementary material for: Global Marine Cold Seep Metagenomes Reveal Diversity of Taxonomy, Metabolic Function, and Natural Products
Source: Genomics Proteomics Bioinformatics. 2023 Dec 13;22(2):qzad006. doi: 10.1093/gpbjnl/qzad006 (PMC12016038; doi:10.1093/gpbjnl/qzad006)
Supplement: qzad006_Supplementary_Data [file qzad006_supplementary_data.zip › Figure S4.pdf]

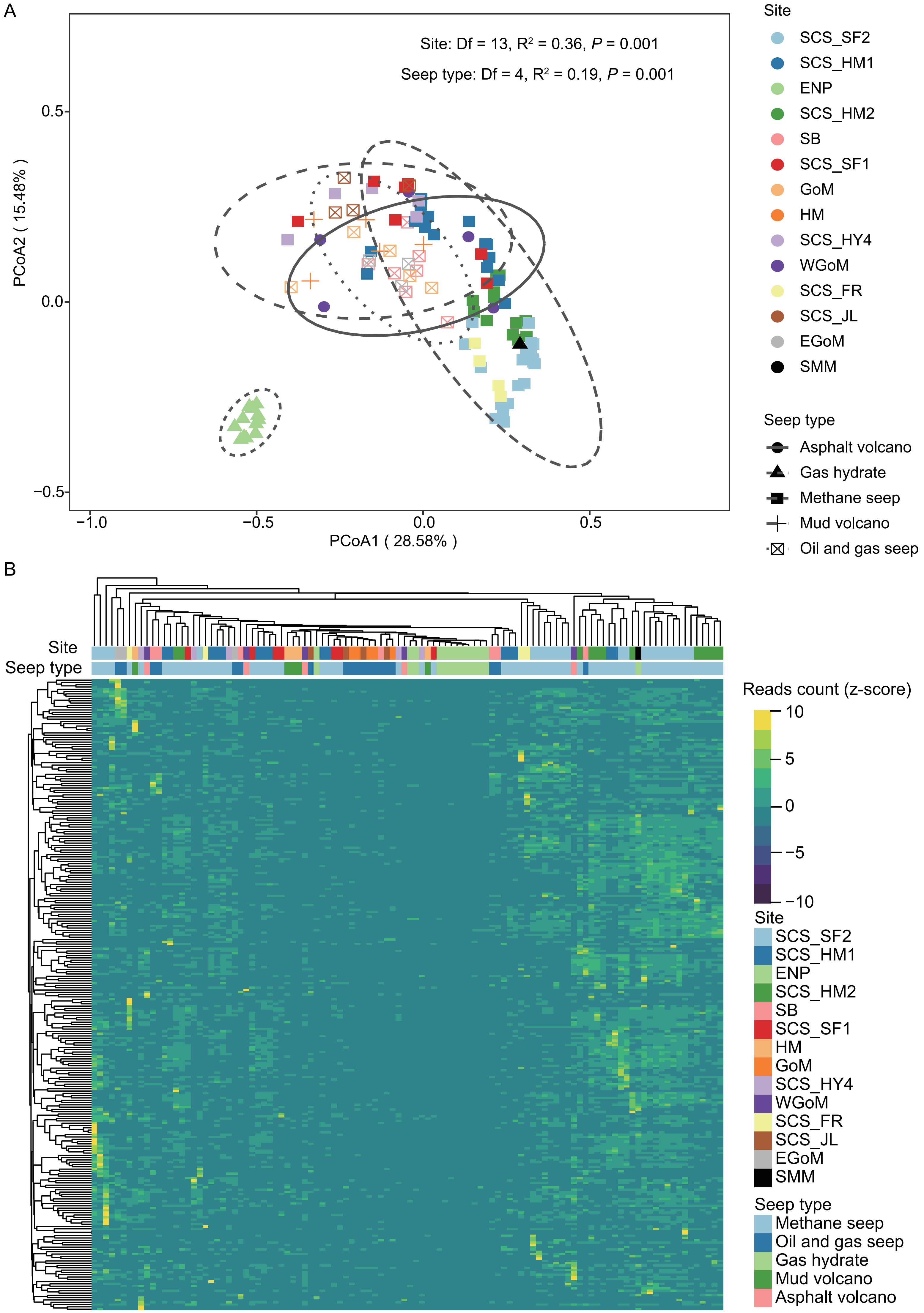
A
Site: Df = 13, R² = 0.36, P = 0.001
Seep type: Df = 4, R² = 0.19, P = 0.001
PCoA2 (15.48%)
PCoA1 (28.58%)
0.5
0.0
−0.5
−1.0
Site
SCS_SF2
SCS_HM1
ENP
SCS_HM2
SB
SCS_SF1
GoM
HM
SCS_HY4
WGoM
SCS_FR
SCS_JL
EGoM
SMM
Seep type
Asphalt volcano
Gas hydrate
Methane seep
Mud volcano
Oil and gas seep
B
Site
Seep type
Reads count (z-score)
10
5
0
−5
−10
Site
SCS_SF2
SCS_HM1
ENP
SCS_HM2
SB
SCS_SF1
HM
GoM
SCS_HY4
WGoM
SCS_FR
SCS_JL
EGoM
SMM
Seep type
Methane seep
Oil and gas seep
Gas hydrate
Mud volcano
Asphalt volcano
