# Supplementary figures and images for: Global Marine Cold Seep Metagenomes Reveal Diversity of Taxonomy, Metabolic Function, and Natural Products
Source: Genomics Proteomics Bioinformatics. 2023 Dec 13;22(2):qzad006. doi: 10.1093/gpbjnl/qzad006 (PMC12016038; doi:10.1093/gpbjnl/qzad006)

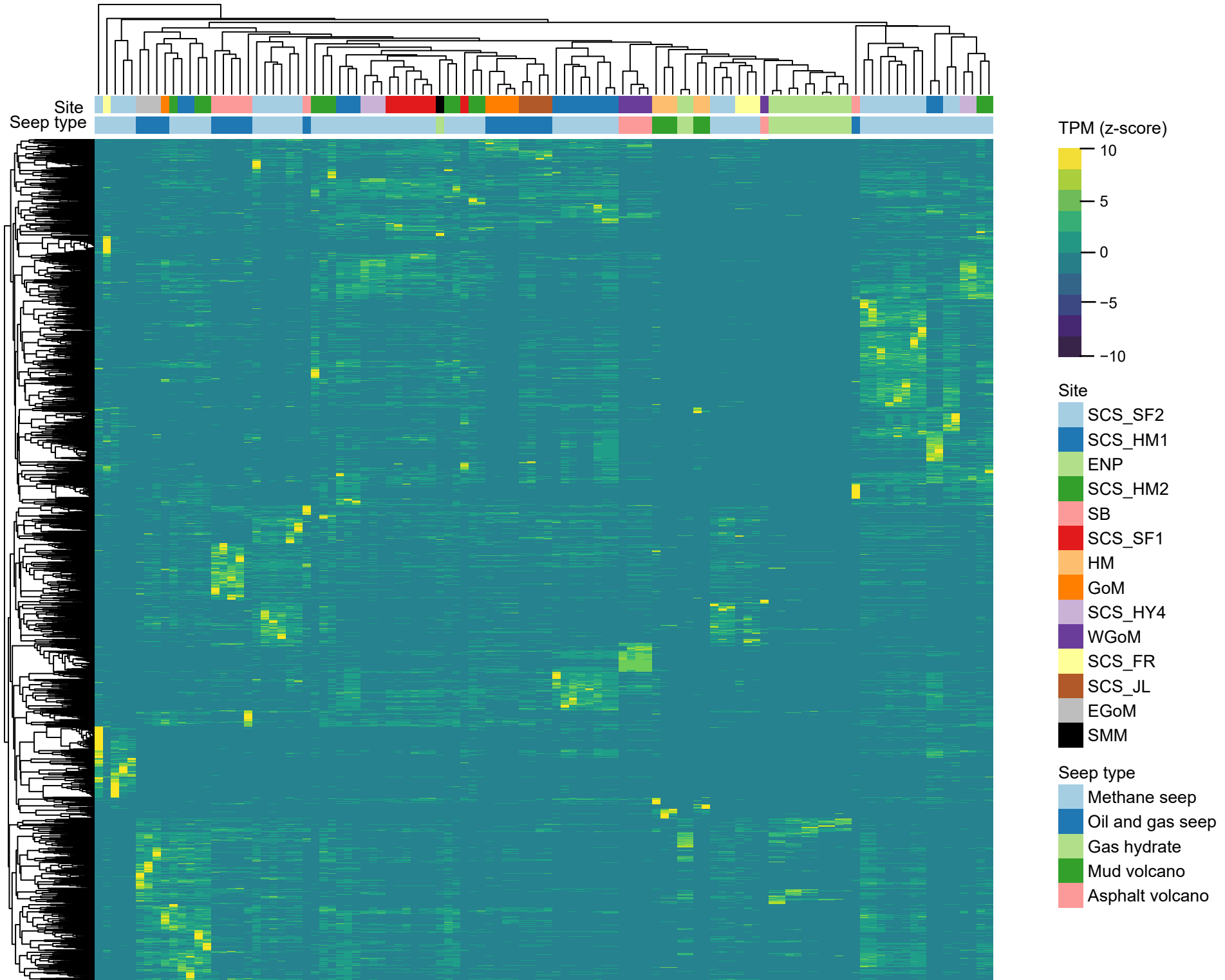

Supplement: qzad006_Supplementary_Data [file qzad006_supplementary_data.zip › Figure S1.pdf]

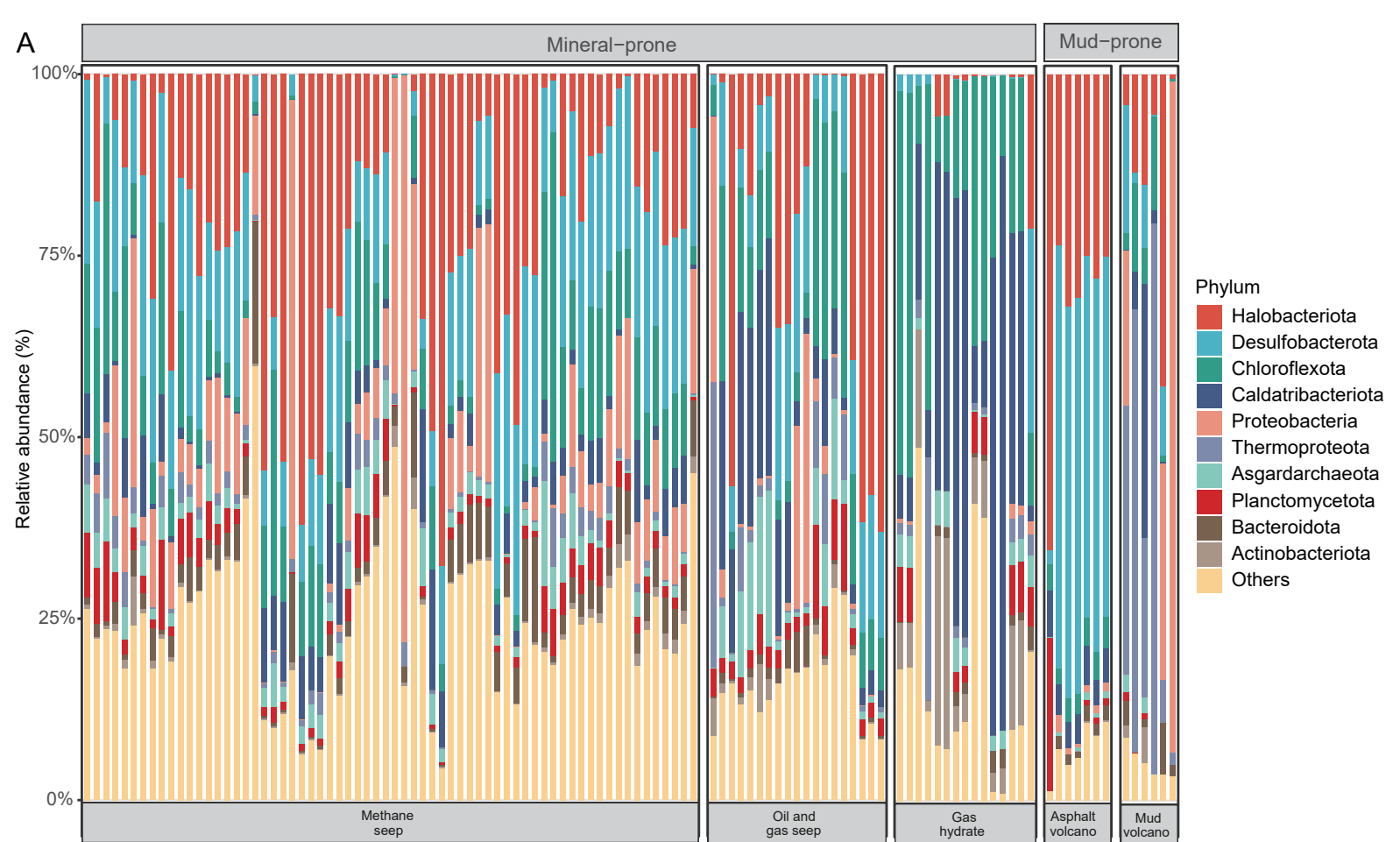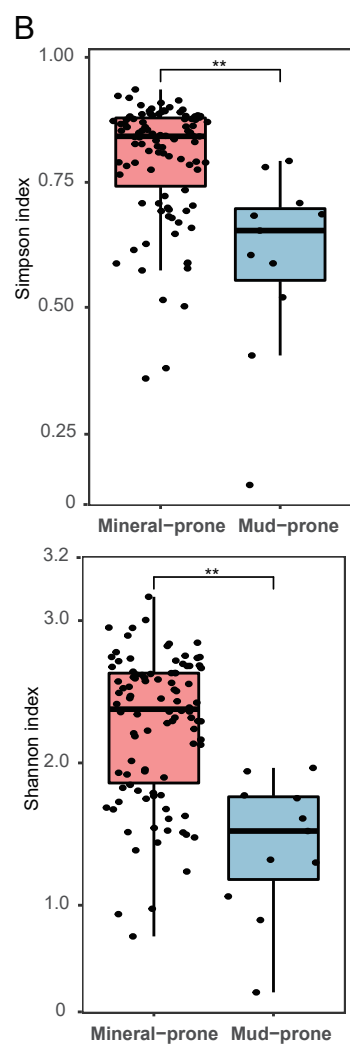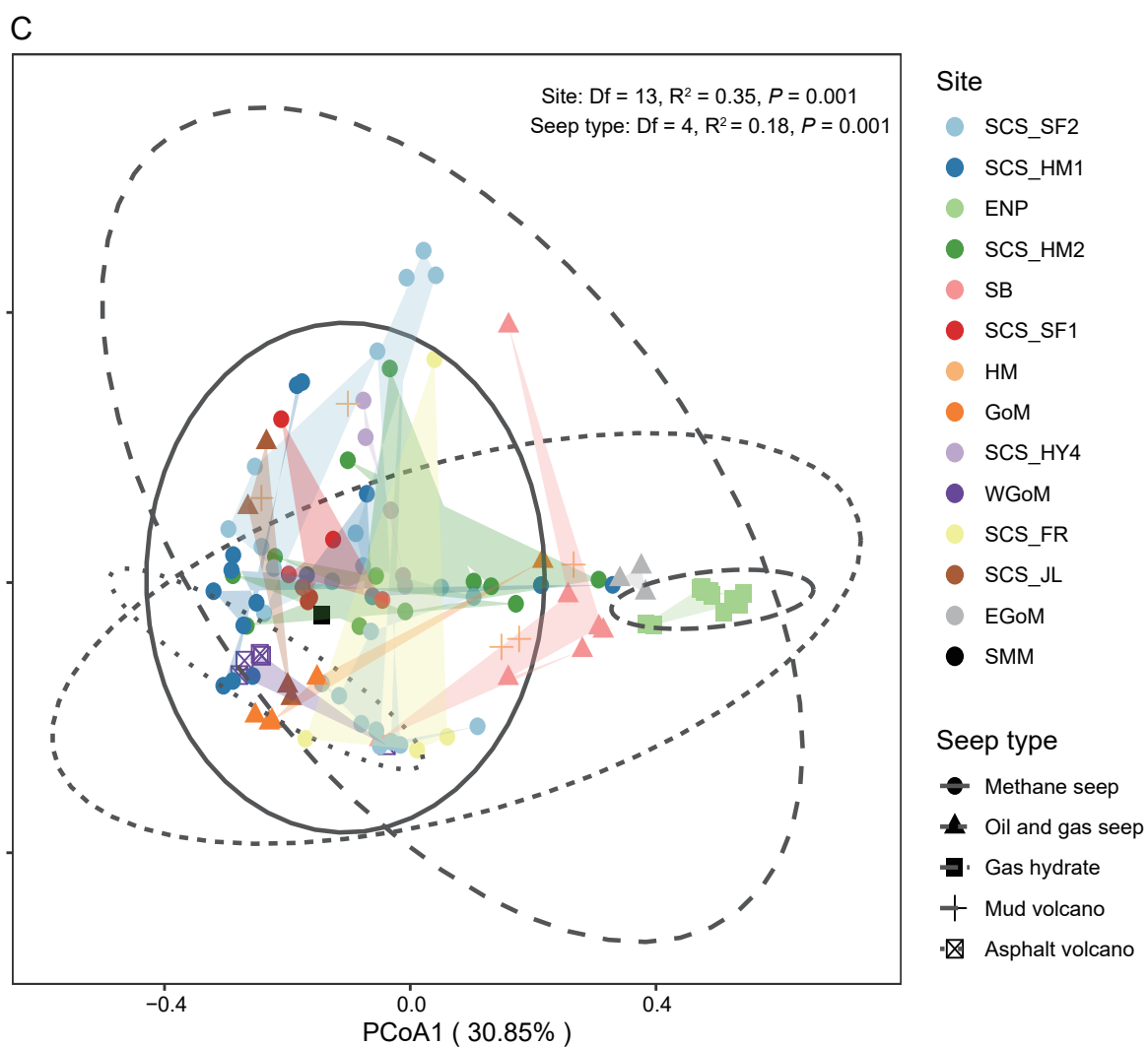

Supplement: qzad006_Supplementary_Data [file qzad006_supplementary_data.zip › Figure S2.pdf]

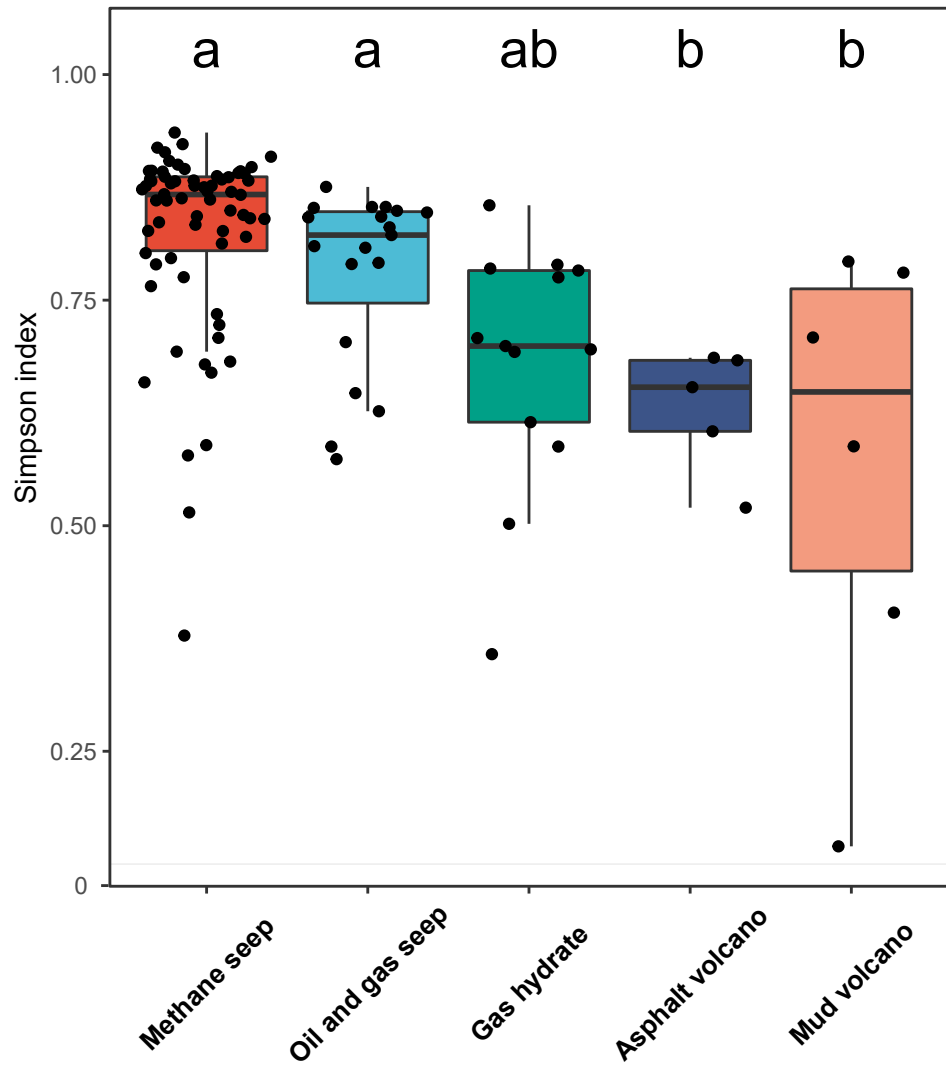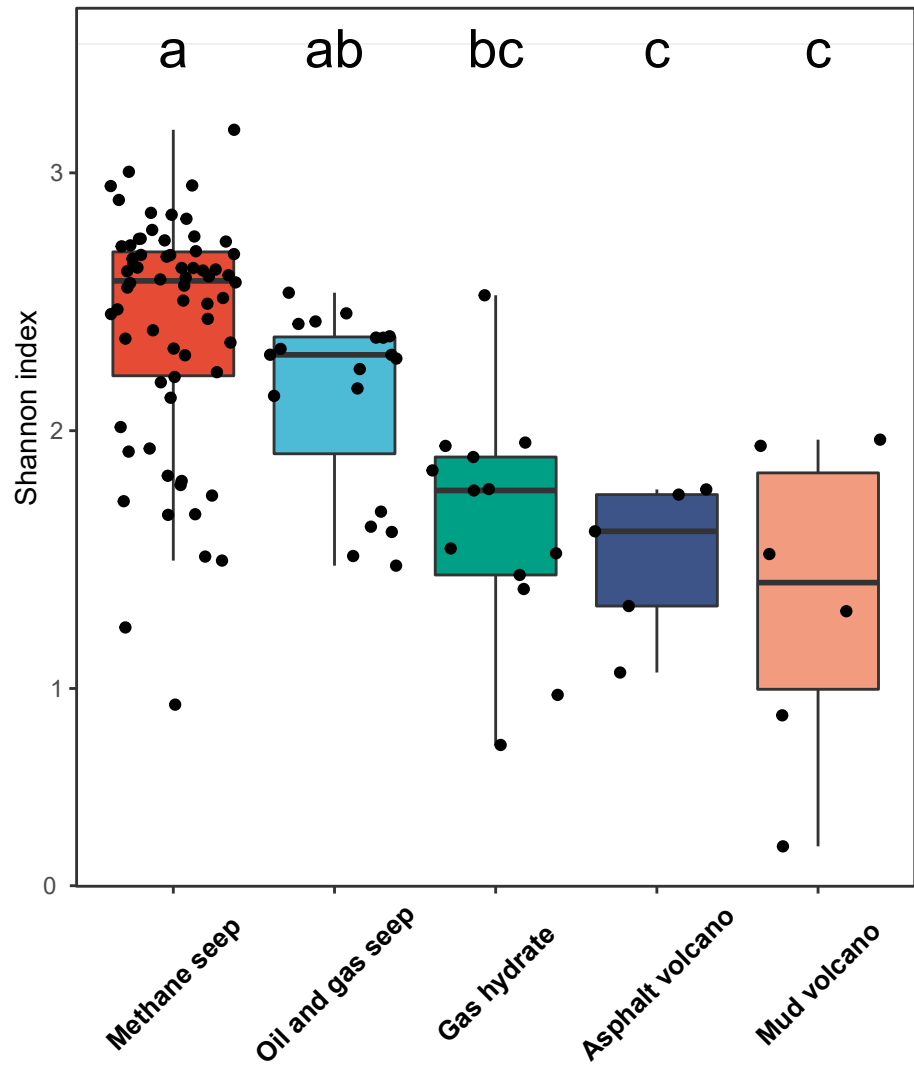

Supplement: qzad006_Supplementary_Data [file qzad006_supplementary_data.zip › Figure S3.pdf]

A

## PKS-NRPS hybrids clusters of MAG SRR13892603\_vb\_S1C4173

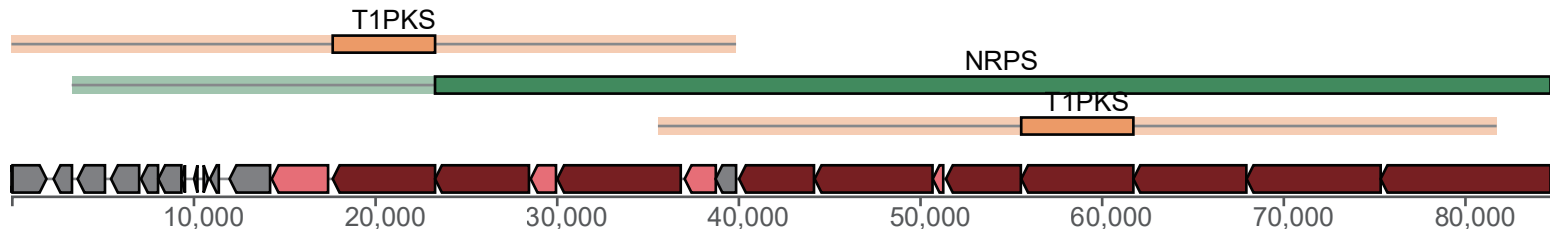

B

## RiPPs cluster of MAG SRR13892601\_vb\_S1C33830

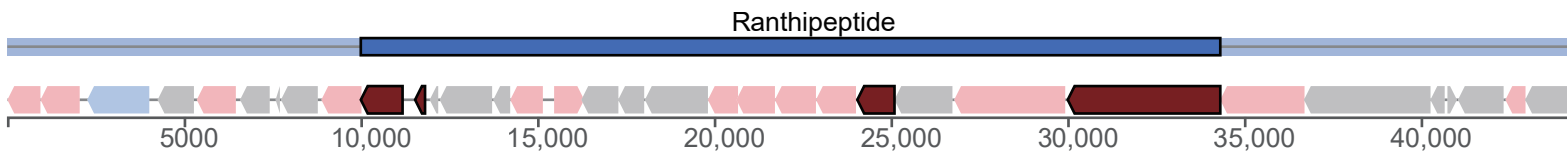

Legend:

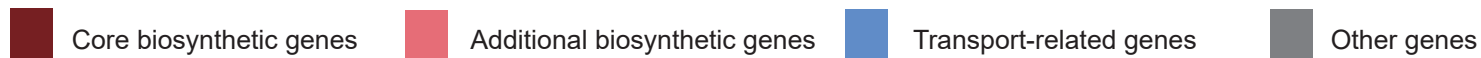

Supplement: qzad006_Supplementary_Data [file qzad006_supplementary_data.zip › Figure S5.pdf]
